# Supplementary material for: ARPES detection of superconducting gap sign in unconventional superconductors
Source: Nat Commun. 2024 May 28;15:4538. doi: 10.1038/s41467-024-48610-9 (PMC11133361; doi:10.1038/s41467-024-48610-9)
Supplement: Supplementary file 1 — Supplementary Information [file 41467_2024_48610_MOESM1_ESM.pdf]

Supplementary Information

ARPES Detection of Superconducting Gap Sign in Unconventional Superconductors

Qiang Gao[1,♯], Jin Mo Bok[2,♯], Ping Ai[1,♯], Jing Liu[1,3,♯], Hongtao Yan[1], Xiangyu Luo[1,4], Yongqing Cai[1], Cong Li[1], Yang Wang[1], Chaohui Yin[1,4], Hao Chen[1,4], Genda Gu[5], Fengfeng Zhang[6], Feng Yang[6], Shenjin Zhang[6], Qinjun Peng[6], Zhihai Zhu[1,4,7], Guodong Liu[1,4,7], Zuyan Xu[6], Tao Xiang[1,3,4], Lin Zhao[1,4,7,*], Han-Yong Choi[8,*] and X. J. Zhou[1,4,7,*]

[1]*Beijing National Laboratory for Condensed Matter Physics, Institute of Physics, Chinese Academy of Sciences, Beijing 100190, China.*

[2]*Department of Physics, Pohang University of Science and Technology (POSTECH), Pohang 37673, Korea*

[3]*Beijing Academy of Quantum Information Sciences, Beijing 100193, China*

[4]*School of Physical Sciences, University of Chinese Academy of Sciences, Beijing 100049, China.*

[5]*Condensed Matter Physics and Materials Science Department, Brookhaven National Laboratory, Upton, New York, 11973, USA*

[6]*Technical Institute of Physics and Chemistry, Chinese Academy of Sciences, Beijing 100190, China.*

[7]*Songshan Lake Materials Laboratory, Dongguan, Guangdong 523808, China.*

[8]*Department of Physics and Institute for Basic Science Research, SungKyunKwan University, Suwon 440-746, Korea*

[♯]*These authors contributed equally: Qiang Gao, Jin Mo Bok, Ping Ai, Jing Liu.*

[*]*Corresponding authors: LZhao@iphy.ac.cn, hychoi@skku.edu, XJZhou@iphy.ac.cn*

(Dated: May 1, 2024)

Supplementary Note 1: Theoretical simulations of a two-band system in the superconducting state.

ARPES measures the single particle spectral function $A(k, \omega)$:

$$I_{ARPES} = I_0 \cdot A(k, \omega) \cdot f(\omega, T) \tag{1}$$

where I_0 is a prefactor and $f(\omega, T)$ is the Fermi distribuction function. The single particle spectral function $A(k, \omega)$ is the imaginary part of the Green's function $G(k, \omega)$:

$$A(k, \omega) = -\frac{1}{\pi} Im[G(k, \omega)] \tag{2}$$

The Green's function is obtained from the Hamiltonian:

$$G(k, \omega) = (\omega - \Sigma(k, \omega) - H)^{-1} \tag{3}$$

where $\Sigma(k, \omega)$ is the electron self-energy.

For a system with two bands (α and β) which are close by in momentum space (Figs. 1(c-h) in the main text), its superconducting state can be described by a phenomenological Hamiltonian

$$H(\mathbf{k}) = \begin{pmatrix} \varepsilon_{i\alpha} & V & \Delta_{i\alpha} & 0 \\ V & \varepsilon_{i\beta} & 0 & \Delta_{i\beta} \\ \Delta_{i\alpha} & 0 & -\varepsilon_{i\alpha} & -V \\ 0 & \Delta_{i\beta} & -V & -\varepsilon_{i\beta} \end{pmatrix} \tag{4}$$

where $\varepsilon_{i\alpha}$ and $\varepsilon_{i\beta}$ represent the initial α and β bare bands, V is the coupling strength between the two bands, and $\Delta_{i\alpha}$ and $\Delta_{i\beta}$ are the initial superconducting gap of the α and β bands. Such a Hamiltonian can also describe the normal state when the initial superconducting gaps are taken as zeros.

The eigenvalues of the above 4×4 matrix (Equation (4)) are

$$E_{\mathbf{k}} = \pm \frac{1}{\sqrt{2}} \sqrt{2V^2 + \Delta_{i\alpha}^2 + \Delta_{i\beta}^2 + \varepsilon_{i\alpha}^2 + \varepsilon_{i\beta}^2 \pm E_\delta} \tag{5}$$

where

$$E_\delta = \sqrt{(\Delta_{i\alpha}^2 - \Delta_{i\beta}^2 + \varepsilon_{i\alpha}^2 - \varepsilon_{i\beta}^2)^2 + 4V^2((\Delta_{i\alpha} - \Delta_{i\beta})^2 + (\varepsilon_{i\alpha} + \varepsilon_{i\beta})^2)} \tag{6}$$

Figures 1c-1i in the main text show the simulated band structures based on the above Equations (2-4) where the initial two bare bands are degenerate. Supplementary Fig. 1

shows the simulated band structures where the initial two bare bands increase in the separation in the momentum space. In both cases, the simulated results of the same gap sign and the opposite gap sign are compared.

Supplementary Note 2: Band structure simulations of Bi2212 in the superconducting state.

It has been well established experimentally that the superstructure bands in Bi2212 are replicas of the main bands, i.e., except for the intensity difference, the dispersion and the superconducting gap of the superstructure bands all mimic those of the main bands. As illustrated in Supplementary Fig. 2, in the case of the d-wave superconducting gap, the gap nodes are along the ΓX and ΓY directions. The presence of a superstructure shifts the original band structures by $\pm n * Q$. For example, a new superstructure Fermi surface is formed by shifting the original main Fermi surface with -Q for $n = -1$ as shown in Supplementary Fig. 2. The superconducting gap remains d-wave for these superstructure Fermi surface, but it is in the shifted Brillouin zone with a new center Γ'. The gap nodes on the superstructure Fermi surface are then along the $\Gamma' X'$ and $\Gamma' Y'$ directions. These are the experimental results that have been well established by the previous ARPES measurements[1].

In our theoretical simulations, we took two steps. The first is the formation of the superstructure bands. In this case, the related information are taken from the experimental results, including the intensity ratio between the main band and the superstructure band, the bare band dispersion, and the superconducting gap on the main Fermi surface and superstructure Fermi surface. The second step is to describe the hybridization between the main band and the superstructure band using the standard two band model (Equation (1) in the main text). We note that this two band model (Equation (1)) does not describe the formation of the superstructure bands. Instead, after the superstructure bands are formed, it describes the band hybridization between the main band and the superstructure band.

In Bi2212, it is observed that the main antibonding band exhibits a strong selective hybridization with the superstructure band of the main bonding band in the second quadrant (Figs. 2 and 3 in the main text)[2]. To describe the band structure, band hybridizations and the superconducting gap structure associated with the two bands, we adopt a similar

Hamiltonian as Equation (4)

$$\hat{H}(\mathbf{k}) = \begin{pmatrix} \varepsilon_{iM} & V & \Delta_{iM} & 0 \\ V & \varepsilon_{iS} & 0 & \Delta_{iS} \\ \Delta_{iM} & 0 & -\varepsilon_{iM} & -V \\ 0 & \Delta_{iS} & -V & -\varepsilon_{iS} \end{pmatrix} \tag{7}$$

Here ε_{iM} and ε_{iS} represent the initial main antibonding band (AB) and superstructure bonding band (BB_SS), V is the coupling strength between the two bands, and Δ_{iM} and Δ_{iS} are the initial superconducting gap of the AB and BB_SS bands. The initial main band is described by the tight binding model[3]:

$$\begin{aligned} \varepsilon_{iM}(\mathbf{k}) &= -2t_1(cosk_x + cosk_y) - 4t_2cosk_xcosk_y - 2t_3(cos2k_x + cos2k_y) \\ &- 4t_4(cos2k_xcosk_y + cosk_xcos2k_y) - T_z(a_0 + (cosk_x - cosk_y)^2/4)) + \mu \end{aligned} \tag{8}$$

in which

$$T_z = \pm\sqrt{t_{bi}^2 + (4t_zcos(k_x/2)cos(k_y/2))^2 + 2t_{bi}4t_zcos(k_x/2)cos(k_y/2)cos(k_z/2)} \tag{9}$$

where the physical meaning of each parameter can be found in Ref.[3]. The sign of T_z in Equation (9) is positive for the main bonding band BB and negative for the main antibonding band AB. The tight binding parameters in Equations (8) and (9) are obtained by fitting the measured Fermi surface and band structures as listed in Supplementary Table. 1, with considering a momentum-dependent renormalization factor $Z = 0.5 - 0.14 * |\cos k_x - \cos k_y|$.

Supplementary Table I: The tight binding parameters from ARPES measurement

t_1 (eV)	t_2/t_1	t_3/t_1	t_4/t_1	t_{bi}/t_1	t_z/t_1	μ	k_z	a_0
0.368	-0.342	0.11	-0.087	0.39	0	0.44	0	0.095

The initial superstructure band of the main bonding band, ε_{iS}, can be obtained from shifting the initial main bonding band in Equations (8) and (9) by the superstructure wavevector **Q** which is **Q**=(0.21, 0.21) for the OD78K Bi2212 sample we measured.

For the initial superconducting gap of the main band, Δ_{iM}, a simple d-wave form is taken: $\Delta_{iM}(\mathbf{k}) = \Delta_0(cosk_x - cosk_y)/2$. Here Δ_0 is obtained from our ARPES measurements

of the superconducting gap on the main antibonding Fermi surface of Bi2212 in the first quadrant and it was found that the gap difference between the main bonding Fermi surface and the main antibonding Fermi surface is small[1]. Δ_{iM} exhibits nodal lines along Γ-X and Γ-Y directions, and changes sign in different parts of the Fermi surface (Fig. 1a in the main text). For the initial superconducting gap of the superstructure bonding band, Δ_{iS}, it can be obtained from the initial superconducting gap of the main band by shifting the momentum with a superstructure wavevector **Q** (as shown by the dashed thin lines in Fig. 4i in the main text).

The Green's function is obtained from the Hamiltonian:

$$G(k,\omega) = (\omega - \Sigma(k,\omega) - H)^{-1} \tag{10}$$

where $\Sigma(k,\omega)$ is the electron self-energy.

$$\Sigma(k,\omega) = \Sigma'(k,\omega) + i\Sigma''(k,\omega) \tag{11}$$

We simulate the spectrum with self-energy of the marginal Fermi liquid to describe the interaction between the electrons[4].

$$\Sigma''(k,\omega) = \lambda\sqrt{\omega^2 + (\pi k_B T)^2} + \Gamma_0 \tag{12}$$

We ignor the real part of the selfenergy and use the tight binding model in Equation (8) to describe the band dispersion. Our simulation well reproduces the electronic structure near the Fermi level. The spectral function is obtained by tracing the imaginary part in the electron channel of the Green's function

$$A(k,\omega) = -\frac{1}{\pi}(Im[G_{11}(k,\omega)] + \kappa{\cdot}Im[G_{22}(k,\omega)]) \tag{13}$$

where κ=0.1 is the intensity ratio between the superstructure band and the main band.

The Bogoliubov band hybridization (Figs. 2h-j in the main text), the band structure evolution with momentum (Figs. 3(e,g) in the main text), the Fermi surface hybridization (Fig. 3(d,f) in the main text) and the unusual momentum dependence of the superconducting gap (Fig. 4i in the main text) in Bi2212 are globally simulated based on the Hamiltonian (Equation (7)) and the above bare bands and initial superconducting gaps with a coupling strength V=12 meV.

Supplementary Note 3: Detailed momentum dependence of the Bogoliubov band hybridization in Bi2212 measured at 15 K in the second quadrant.

Supplementary Fig. 3 shows the detailed momentum dependence of the Bogoliubov band hybridization. Near the nodal region, the main antibonding band is mainly observed (Cut1 to Cut3). Away from the nodal region, in the crossing area of the main antibonding Fermi surface and the superstructure bonding Fermi surface, strong Bogoliubov hybridization occurs between the main antibonding band and the superstructure bonding band (Cut4 to Cut8). When the momentum cuts move toward the antinodal region, the Bogoliubov hybridization gets weaker and becomes invisible (Cut9 to Cut14).

To quantitatively determine the Bogoliubov band hybridization, we extract the hybridization gap Δ_h. Supplementary Figs. 4a and 4b show the momentum dependence of the band structures near the crossing area. As shown in Supplementary Fig. 4d, the Bogoliubov band hybridization gap can be determined by the distance of the peaks in the EDCs at k_H. The obtained Bogoliubov band hybridization gap as a function of momentum is shown in Supplementary Fig. 4e.

Supplementary Note 4: Momentum-dependent band structures and superconducting gap of Bi2212 in the first quadrant.

Supplementary Fig. 5 shows the momentum-dependent band structures of Bi2212 (OD78K) in the first quadrant measured at 15 K. Due to the photoemission matrix element effects, the main AB band is predominant and the main BB band becomes visible near the antinodal region. No obvious Bogoliubov band hybridization is observed between the AB and BB bands.

Supplementary Fig. 6 shows the EDCs (Supplementary Fig. 6b) and symmetrized EDCs (Supplementary Fig. 6c) along the antibonding Fermi surface (AB). The obtained momentum dependent superconducting gap is shown in Supplementary Fig. 6d. It can be fitted by a d-wave gap form $\Delta = 29 * |cosk_x - cosk_y|/2$.

Supplementary Note 5: Unusual gapless superconductor with zero gap on the entire Fermi surface.

Our simulations indicate that some unusual superconductors may be designed and produced. As shown in Supplementary Fig. 7, we consider a material that consists of two Fermi

surface sheets. These two Fermi surface (Supplementary Fig. 7d) are produced from the initial degenerate bands (Supplementary Figs. 7(a,b)) and the interband interaction. If the superconducting gap sign is opposite on the two bands, it is possible that the superconducting gap becomes zero on both Fermi surface sheets even though the initial gap is non-zero (Supplementary Figs. 7(e,f)). This would produce a superconductor with zero superconducting gap on the entire Fermi surface. It is interesting to explore whether such unusual superconductors can be realized both theoretically and experimentally.

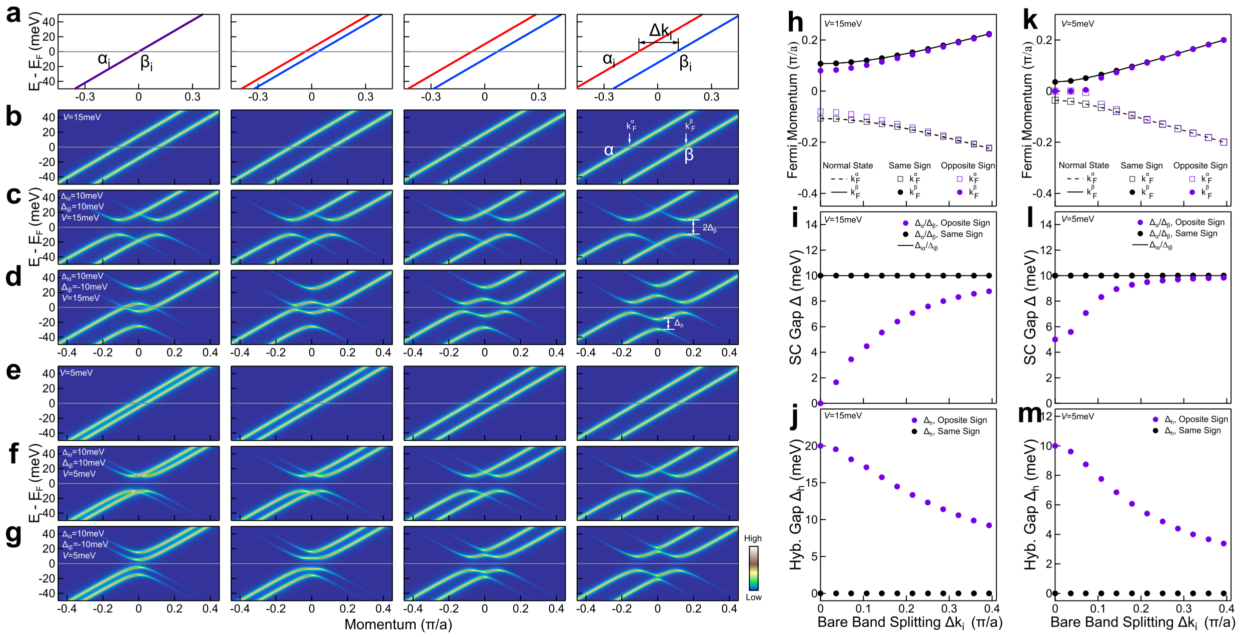

Supplementary Fig. 1: **Bogoliubov band hybridizations in the simulated band structures with different bare bands. a**. Different initial bare bands (α_i and β_i) from degenerate (leftmost panel) to nondegenerate cases (right panels). The difference between the two bare bands is represented by the momentum seperation Δ_k as marked by the rightmost panel. **b,c,d**. Evolution of the simulated band structures with the bare bands after putting the interband coupling (V=15 meV). **b** shows the simulated α and β bands in the normal state. **c** shows the simulated band structures in the superconducting state. Here the same magnitude (Δ_i=10 meV) and the same sign of the initial superconducting gaps are taken for the two bands. **d**, Same as **c** but the opposite sign of the initial superconducting gaps are taken for the two bands. **e,f,g**, Same as **b,c,d** but with a smaller interband coupling V=5 meV. **h,i,j**. Evolution of the Fermi momenta of the α and β bands (k_F^α and k_F^β) (**h**), the superconducting gap Δ_α and Δ_β (**i**) and the hybridization gap Δ_h (**j**) with the Fermi momentum separation Δ_k of the initial two bare bands. They are obtained from **b-d** with the interband coupling V=15 meV. **k-m**. Same as **h-j** but obtained from **e-g** with an interband coupling V=5 meV.

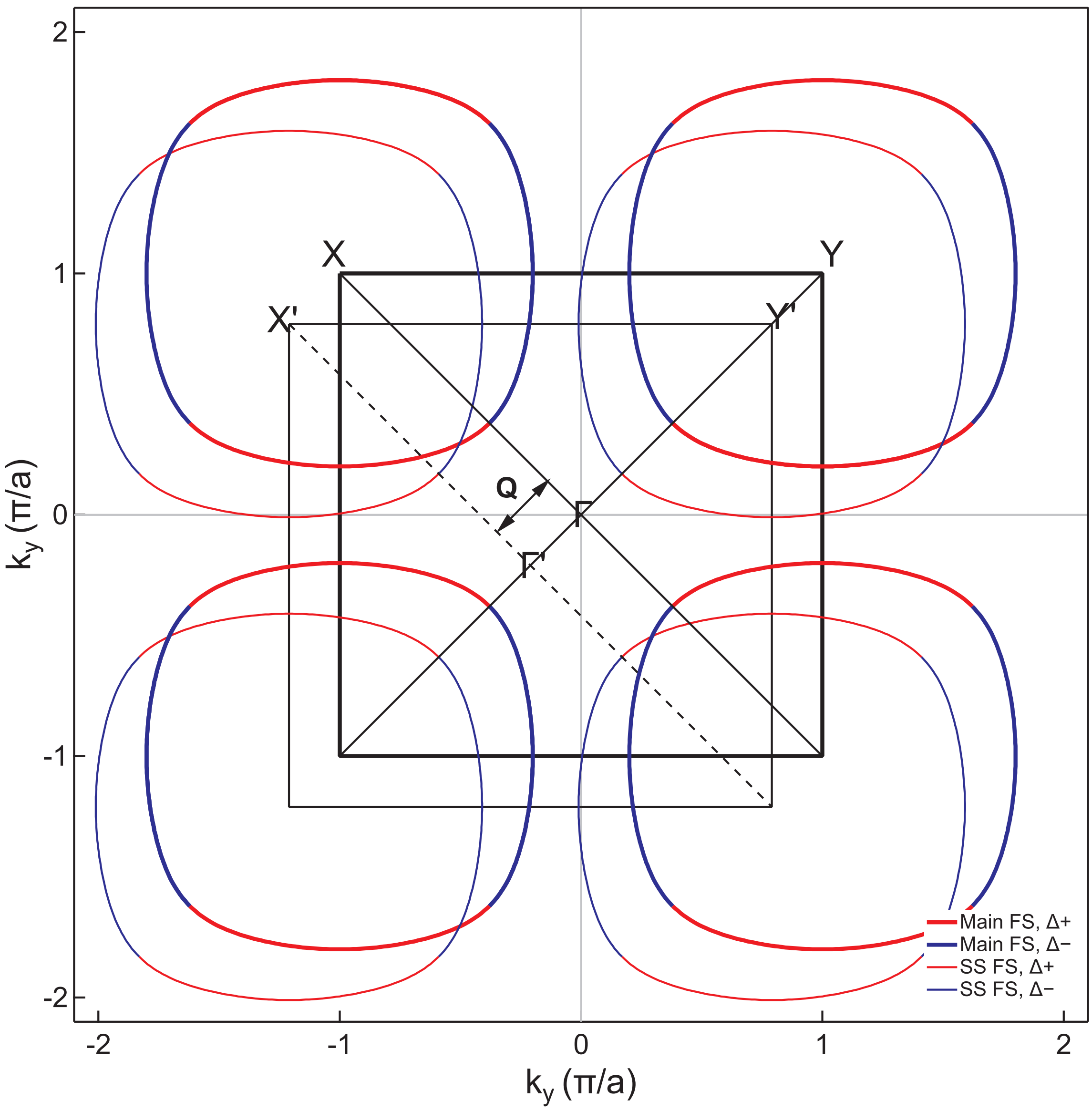

Supplementary Fig. 2: **Schematic Fermi surface of Bi2212.** The main bonding Fermi surface (thick blue and red lines) and the superstructure bonding Fermi surface (thin blue and red lines) are plotted. The superstructure Fermi surface is obtained by shifting the main Fermi surface with a superstructure wave vector **Q** along ΓY direction. The blue and red lines represent positive and negative sign of the superconducting gap, respectively.

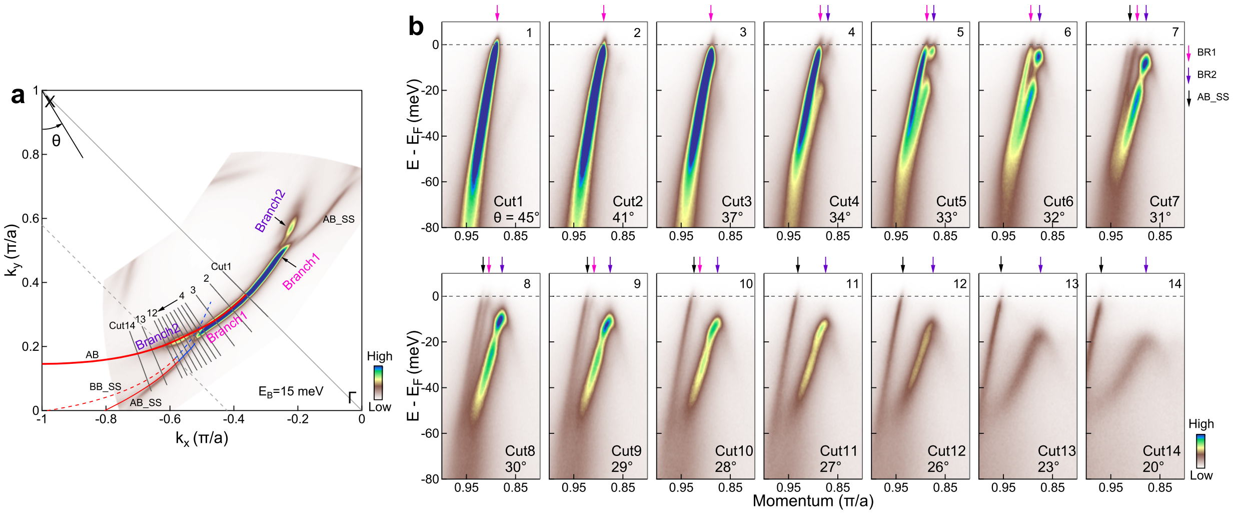

Supplementary Fig. 3: **Detailed momentum dependence of the Bogoliubov band hybridization in Bi2212.** **a**, Constant energy contour at the binding energy of 15 meV measured at 15 K in the second quadrant. The data are symmetrized with respect to the Γ-X nodal line. Due to the photoemission matrix element effects, the main AB Fermi surface and its superstructure Fermi surface AB_SS are observed. The main AB Fermi surface is broken into two branches (Branch1 and Branch2) due to the hybridization with the superstructure BB_SS Fermi surface. **b**, Band structures measured along different momentum cuts from the nodal direction to the antinodal region. The location of the momentum cuts is shown by the black lines in **a**. These momentum cuts all point to X point so their locations are also marked by the Fermi surface angle θ, as defined in **a**. Three bands are observed corresponding to Branch1, Branch2 and AB_SS as marked by the coloured arrows and labelled as BR1, BR2 and AB_SS on top of each panel in **b**. The Bogoliubov band hybridization occurs between the BR1 and BR2 bands which is clear in the measurements of Cut4-Cut11.

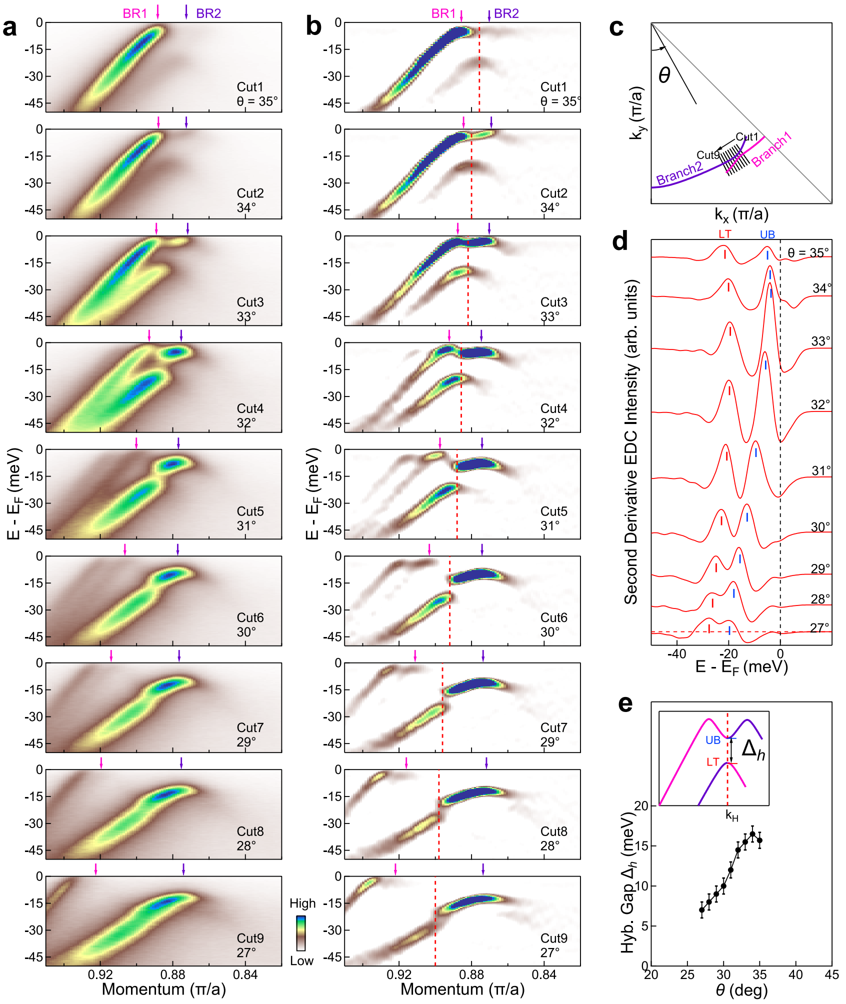

Supplementary Fig. 4: **Determination of the momentum-dependent Bogoliubov band hybridization gap in Bi2212.** a, Momentum-dependent band structures measured at 15 K in the second quadrant. The location of the momentum cuts is shown by the solid black lines in **c**, also marked by the corresponding Fermi surface angle θ. **b**, Corresponding second-derivative images of **a** with respect to energy. The two related bands, BR1 and BR2, corresponding to Branch1 and Branch2 Fermi surface in **c**, are marked by pink arrows and purple arrows, respectively, in **a** and **b**. The momentum location of the red dashed lines in **b** represents the hybridization momentum k_H as schematically shown in the inset of **e**. **c**, Second quadrant with the related Branch1 and Branch2 Fermi surface schematically shown. **d**, Second-derivative EDCs at the hybridization momentum obtained from **b**. Two peaks are observed in each EDC marked by blue and red ticks. As seen in the inset of **e**, the blue tick corresponds to the energy position of the local bottom of the upper band (UB) while the red tick corresponds to the energy position of the local top of the lower band (LT). The energy difference between the two peaks in each EDC in **d** corresponds to the hybridization gap Δ_h. **e**, The momentum dependence of the Bogoliubov hybridization gap Δ_h obtained from **d**. Error bars are determined based on the fitting error of the EDC peak position.

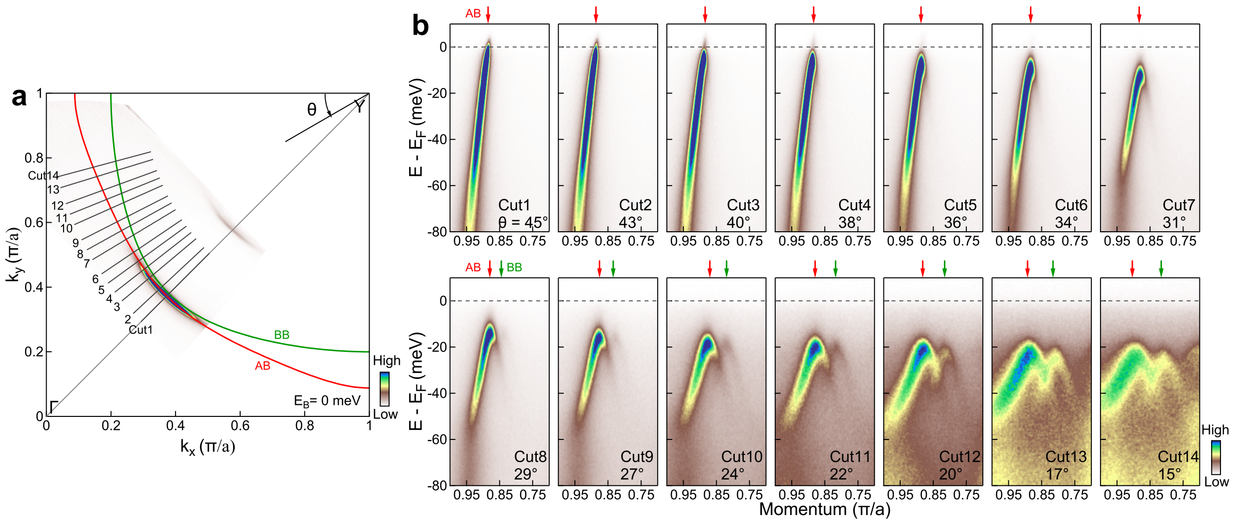

Supplementary Fig. 5: **Momentum dependent band structures of Bi2212 measured at 15 K in the first quadrant.** **a**, Fermi surface mapping of Bi2212. The two main Fermi surface, AB and BB, are plotted as guidelines. **b**, Band structures measured along different momentum cuts from the nodal direction to the antinodal region. The location of the momentum cuts is shown by the black lines in **a**. These momentum cuts all point to the Y point so their locations are also marked by the Fermi surface angle θ, as defined in **a**. Due to the photoemission matrix element effects, the main AB band is predominant in the spectral intensity although the main BB band becomes visible near the antinodal region as marked by the coloured arrows. No obvious Bogoliubov band hybridization is observed between the AB and BB bands.

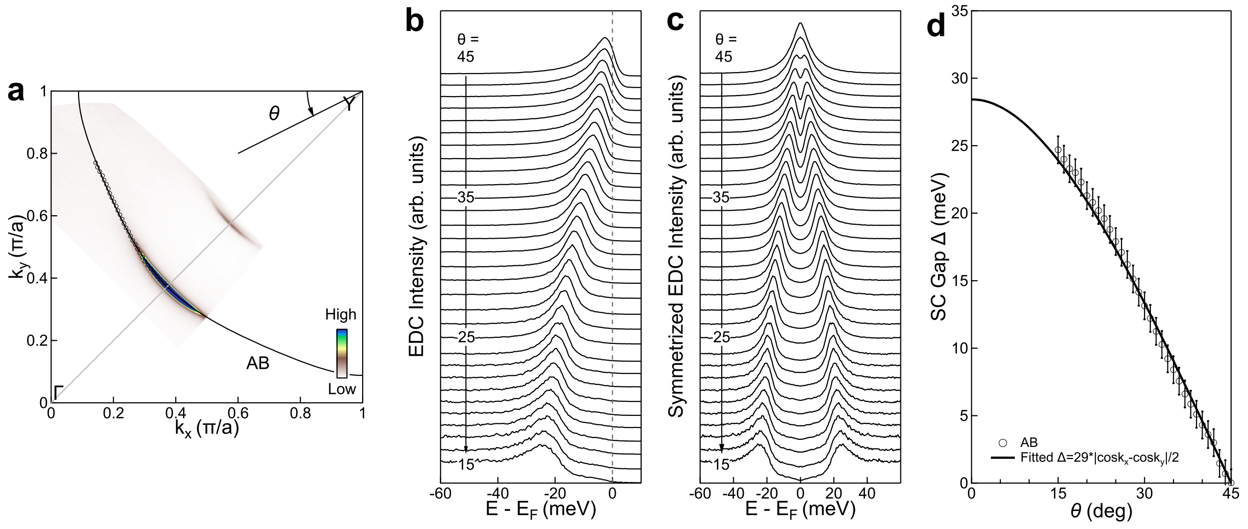

Supplementary Fig. 6: **Momentum-dependent superconducting gap of Bi2212 in the first quadrant.** **a**. Fermi surface mapping of Bi2212 in the first quadrant measured at 15 K. The antibonding Fermi surface (AB) is mainly observed. **b**. EDCs on the AB Fermi surface in the first quadrant. The location of the Fermi momentum points is shown by the black open circles in **a**. It is also defined by the Fermi surface angle θ as shown in **a**. **c**. The corresponding symmetrized EDCs obtained from **b**. **d**. Momentum dependent superconducting gap (black open circles) along the AB Fermi surface obtained from the symmetrized EDCs in **c**. The black solid line represents the fitted d-wave superconducting gap $\Delta = 29 * |cosk_x - cosk_y|/2$.

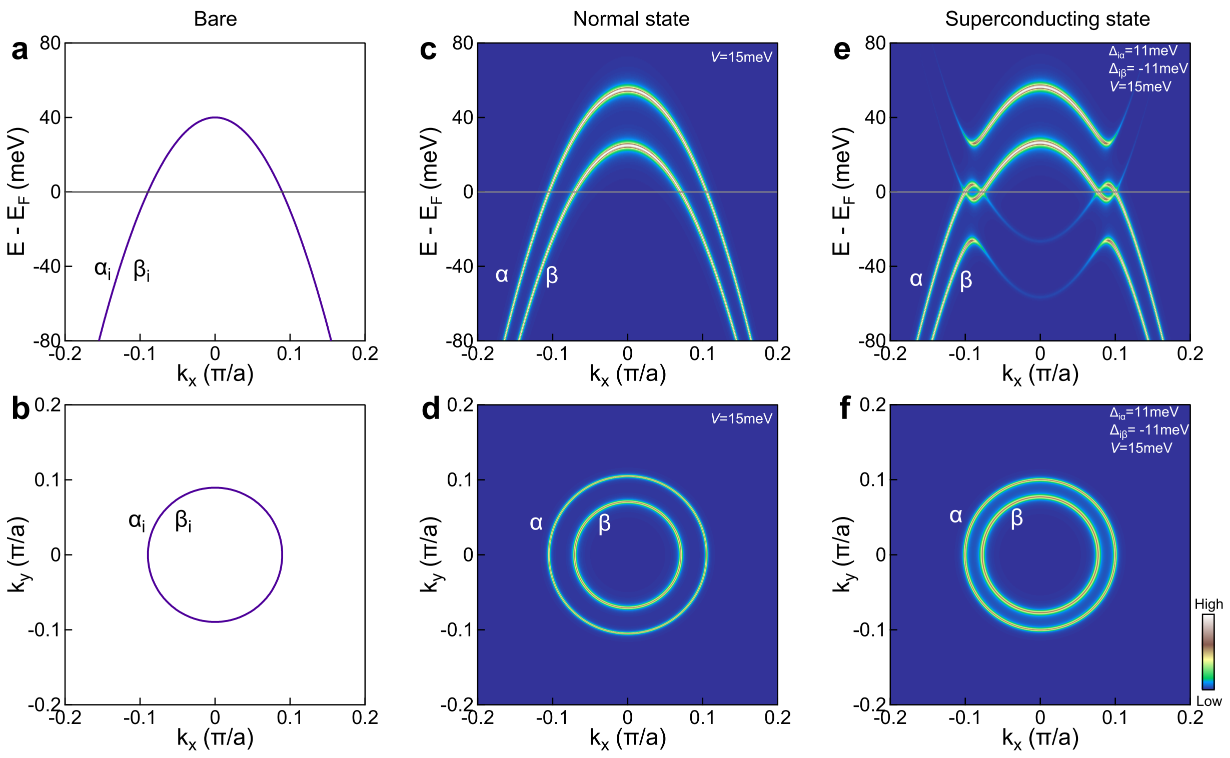

Supplementary Fig. 7: **Unconventional superconductor with gapless Fermi surface.** **a**, Initial bare bands (α_i and β_i) which are degenerate. **b**, Initial Fermi surface (α_i and β_i). **c**, Simulated band structure in the normal state after putting the interband coupling (V=15 meV). **d**, The corresponding simulated Fermi surface (α and β) in the normal state. **e**, Simulated band structure in the superconducting state. Here the same magnitude (Δ_i=11 meV) and the opposite sign of the initial superconducting gaps are taken for the two bands. There are bands crossing Fermi level with zero gap. **f**, The corresponding simulated Fermi surface (α and β) in the superconducting state.

Supplementary references

---

[1] Ai, P. et al. Distinct Superconducting Gap on Two Bilayer-Split Fermi Surface Sheets in $Bi_2Sr_2CaCu_2O_{8+\delta}$ Superconductor. *Chinese Physics Letters* **36**, 067402 (2019).

[2] Gao, Q. et al. Selective hybridization between the main band and the superstructure band in the $Bi_2Sr_2CaCu_2O_{8+\delta}$ superconductor. *Physical Review B* **101**, 014513 (2020).

[3] Markiewicz, R. S. et al. One-band tight-binding model parametrization of the high-Tc cuprates including the effect of kz dispersion. *Physical Review B* **72**, 054519 (2005).

[4] Bok, J. M. et al. Quantitative determination of pairing interactions for high-temperature superconductivity in cuprates. *Science Advances* **2**:e1501329 (2016).
